# HLA-DRB1*04:05 is involved in the development of Vogt–Koyanagi–Harada disease-like immune-related adverse events in patients receiving immune checkpoint inhibitors

**DOI:** 10.1038/s41598-023-40565-z

**Published:** 2023-08-21

**Authors:** Masaki Takeuchi, Akira Meguro, Jutaro Nakamura, Rei Chikagawa, Raiga Osada, Etsuko Shibuya, Yukiko Hasumi, Norihiro Yamada, Mami Ishihara, Nobuhisa Mizuki

**Affiliations:** Department of Ophthalmology and Visual Science, Yokohama City University Graduate School of Medicine, 3-9 Fukuura, Kanazawa-ku, Yokohama, 236-0004 Japan

**Keywords:** Immunogenetics, Cancer immunotherapy, Uveal diseases

## Abstract

Immune checkpoint inhibitors (ICIs) activate anti-tumor activity by inhibiting immune checkpoint molecules that suppress inflammatory T-cell activity. However, ICIs can initiate excessive immune responses, thereby causing immune-related adverse events (irAEs). ICI-associated uveitis (ICIU) is an irAE that affects the eyes. Although Vogt–Koyanagi–Harada disease (VKH)-like uveitis is a common form of ICIU, it is unclear which factors determine the ICIU form. We retrospectively reviewed the medical records of nine ICIU cases treated with ICIs for malignancies. We also performed HLA typing in seven cases to investigate the association between HLA and disease type. Fisher's exact test was used for the statistical analysis. Five of the ICIU cases were VKH-like ICIUs, and four were non-VKH-like ICIUs. No association was found between mean age, sex, primary disease, ICI, time to onset, and disease type. Four patients with VKH-like uveitis underwent HLA genotyping and were all positive for HLA-DRB1*04:05. All 3 patients with non-VKH-like uveitis were negative for HLA-DRB1*04:05. Statistical analysis showed that HLA-DRB1*04:05 was significantly associated with developing VKH-like ICIU (*P* = 0.029). In ICIU, HLA-DRB1*04:05 was associated with the pathogenesis of VKH-like uveitis, suggesting that ICI-associated VKH-like uveitis has a similar pathogenesis to VKH.

## Introduction

Immune checkpoint molecules are involved in the mechanisms of the immunosuppressive function of T-cells. The binding of immune checkpoint molecules, expressed on the surface of activated T-cells, to their ligands on antigen-presenting cells suppresses T-cell activation^1^. Cancer cells escape host immunity by expressing ligands for immune checkpoint molecules on their cell surface. Immune checkpoint inhibitors (ICIs) exert anti-tumor effects by inhibiting the immunosuppression through interaction with immune checkpoint molecules and their ligands. ICIs developed for treating progressive malignancies include PD-1 inhibitors such as nivolumab and pembrolizumab, PD-L1 inhibitors, for example, atezolizumab and durvalumab, and ipilimumab, a CTLA-4 inhibitor.

Despite the efficacy of ICIs, exaggerated immune responses due to the inhibition of immunosuppressive mechanisms can cause immune-related adverse events (irAEs), such as autoimmune diseases. The incidence of irAEs in ICI-treated patients is estimated to be 60–85% and they include skin lesions and GI toxicity. Uveitis is an ICI-induced ocular irAE occurring in about 0.3–1% of ICI-treated patients^2,3^. ICI-associated uveitis (ICIU) can present as anterior uveitis, posterior uveitis, or pan uveitis. Many cases of posterior and pan uveitis have presented with a Vogt–Koyanagi–Harada disease (VKH)-like clinical picture characterized by choroidal thickening and serous subretinal fluid^4^. However, the pathogenesis of ICIU and its differentiating characteristics have not been clarified. In this study, we investigated the background, clinical manifestations, and HLA type of patients who developed noninfectious uveitis during ICI treatment.

## Subject and methods

We retrospectively reviewed the medical records of nine consecutive ICI-treated cancer patients with ICIU diagnosed at the Yokohama City University Hospital between January 2019 and December 2022. We included all patients with ICIU, regardless of the type of malignancy or causative ICI. To diagnose VKH-like uveitis, we used the criteria for ophthalmologic lesions of the Revised Diagnostic Criteria for VKH Disease^5^.

This study was conducted according to the tenets of the Declaration of Helsinki. The Institutional Review Board of Yokohama City University Hospital approved the research protocol and informed consent. Written informed consent was obtained from all the study subjects.

The patients’ age, sex, ICI used, duration of ICI administration until onset, primary disease, other irAEs, clinical manifestations of uveitis, and best-corrected visual acuity (BCVA) at the first and last visits were extracted from the medical records. The BCVA was expressed as a conversion from decimal visual acuity to the log of minimum angle of resolution (logMAR). The haplotype frequencies of HLA-C-HLA-DRB1-HLA-DQB1 in the Japanese population were investigated from the data of 2,938 individuals in the HLA Laboratory database (https://hla.or.jp/)^6^.

Correlations between the disease form of ICIU and age, and BCVA at the first and last visit were statistically analyzed using the Mann–Whitney U test. The BCVA of the right eye in Case 6 was excluded from statistical analysis because of amblyopia. A two-tailed Fisher's exact test was used to analyze the association between disease type and sex, ICI, primary disease, affected eye, anatomic classification, ICI interruption, and HLA type. The significance level was taken as *P* < 0.05. IBM SPSS Statistics ver. 28.0.1.0 (IBM Japan, Ltd., Tokyo, Japan) was used for statistical analysis.

### Ethical approval

This study was conducted in accordance with the tenets of the Declaration of Helsinki. The Institutional Review Board of Yokohama City University Hospital approved the research protocol and informed consent.

### Consent to participate

Written informed consent was obtained from all study subjects to participate in this study and to publish the information and images in an online open-access publication.

## Results

The patient backgrounds are shown in Table 1. There were 9 patients in total, 4 males and 5 females, with a mean age of 65.9 ± 9.4 years. Cases 5 and 8 had a history of VKH before ICI administration (37 and 6 years ago, respectively). The mean time from ICI administration to the onset of uveitis was 74.8 ± 39.6 days, and the mean observation period was 12.7 ± 8.7 months. The primary disease was malignant melanoma in five patients, small-cell lung cancer in two, renal cancer in one, and a combination of gastric cancer and renal cancer in one. Five patients received nivolumab and one received pembrolizumab (PD-1 inhibitors), one received durvalumab and one received atezolizumab (PD-L1 inhibitors). Ipilimumab, (CTLA-4 inhibitor) was administered as combination therapy with nivolumab in one case.

**Table 1:** Summary of clinical manifestations of patients with immune checkpoint inhibitor -associated uveitis.

Case	Age/sex	Primary disease	ICI	Days until onset	Affected eye	ICIU	ICIU form	IrAEs	ICI discontinuation	Treatment other than eye drops	Prognosis
Case 1	71/F	Malignant melanoma	Pembrolizumab	141	Bilateral	Posterior	VKH-like	Interstitial lung disease, thyroid dysfunction	Discontinuation	Sub-tenon injection of triamcinolone acetonide Oral predonisolone 30 mg	Remission
Case 2	71/M	Malignant melanoma	Nivolumab	125	Bilateral	Pan	Nonspecific	–	Discontinuation	Sub-tenon injection of triamcinolone acetonide	Improvement
Case 3	69/F	Renal cancer	Nivolumab	Unknown	Bilateral	Pan	VKH-like	–	Continuation	Sub-conjuctive injection of dexamethasone	Remission
Case 4	72/M	Malignant melanoma	Nivolumab	89	Bilateral	Pan	Nonspecific	Colitis	Continuation	Sub-tenon injection of dexamethasone	Remission
Case 5	60/F	Small cell lung cancer	Durvalumab	68	Bilateral	Posterior	VKH-like	–	Discontinuation	Sub-tenon injection of dexamethasone	Remission
Case 6	66/M	Small cell lung cancer	Atezolizumab	39	Bilateral	Pan	Nonspecific	–	Discontinuation	None	Improvement
Case 7	49/F	Malignant melanoma	Nivolumab	77	Bilateral	Pan	Nonspecific	Colitis, liver dysfunction	Discontinuation	Sub-tenon injection of triamcinolone acetonide	Remission
Case 8	54/F	Gastric cancer and renal cancer	Nivolumab	30	Bilateral	Pan	VKH-like	–	Discontinuation	Methylprednisolone 500 mg for 3 days	Remission
Case 9	81/M	Malignant melanoma	Nivolumab + Ipilimumab	29	Bilateral	Pan	VKH-like	–	Continuation	Sub-tenon injection of triamcinolone acetonide	Remission

Case 1, 4, and 6 died at 10, 22, and 14 months, respectively, after administration of the immune checkpoint inhibitor from progression of the primary disease.

*ICIU* immune checkpoint inhibitor-associated uveitis, *irAE* immune-related adverse event, *VKH-like* Vogt–Koyanagi–Harada disease-like, *Post* posterior uveitis, *Pan* panuveitis.

**Figure 1:**
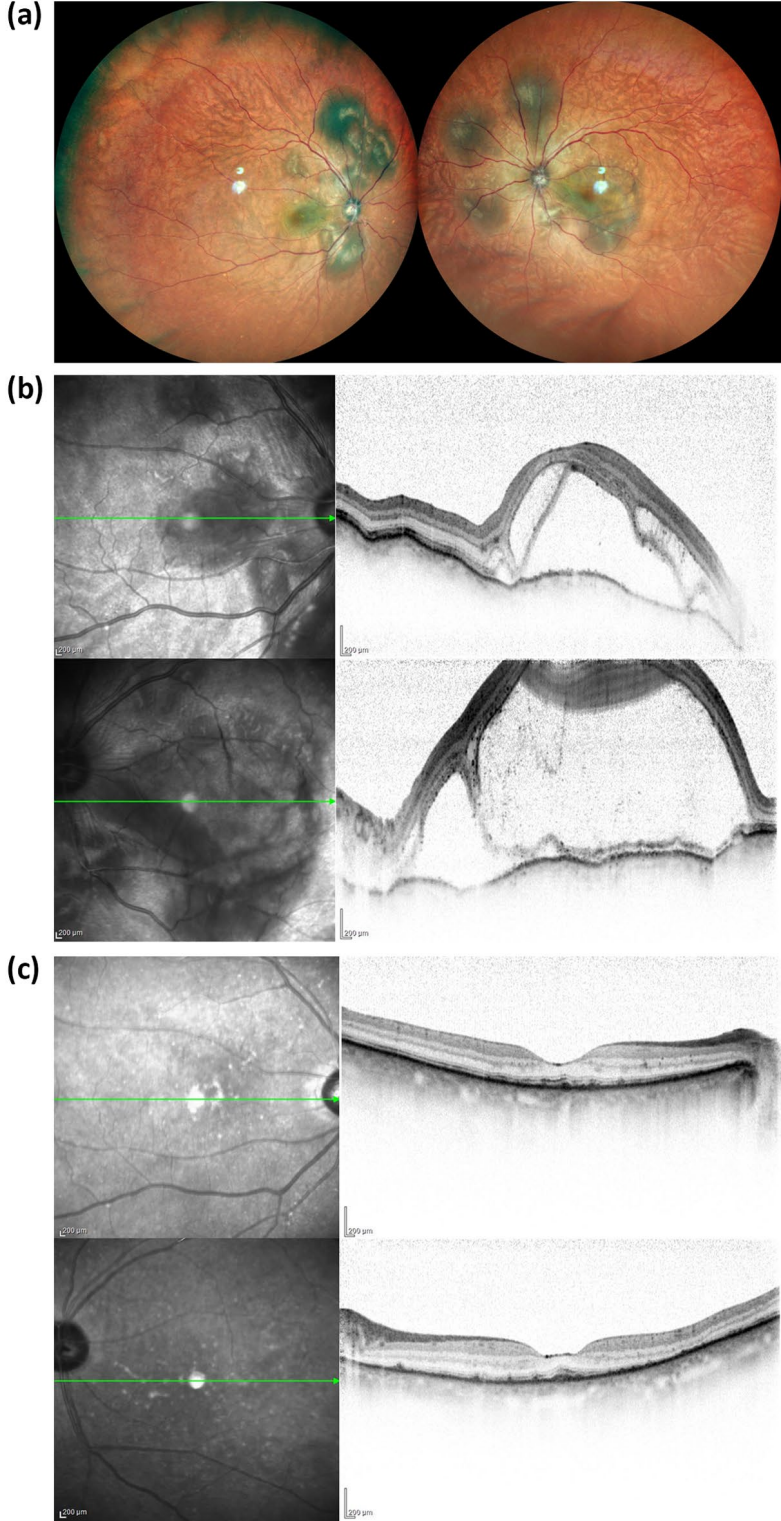
Ophthalmologic findings in a representative case of VKH-like immune checkpoint inhibitor-associated uveitis (Case 1). A 71-year-old female was diagnosed with nasal malignant melanoma with multiple metastases at the age of 70 years. Following heavy particle radiation therapy, pembrolizumab treatment was initiated. Two months after starting immune checkpoint inhibitor (ICI) therapy, she developed grade 1 hyperthyroidism. Five months later, she visited an ophthalmologist due to a decline in visual acuity. Her visual acuity was Vd = (0.3) and Vs = (0.3). Multifocal exudative retinal detachment was observed in both fundi (**a**). Optical coherence tomography revealed an undulating bilateral retinal pigment epithelium, subretinal fluid, and diffuse choroidal thickening (**b**). Concurrently with ocular symptoms, she also reported hearing impairment. Cerebrospinal fluid examination revealed a cerebrospinal fluid cell count of 2/μL. On the basis of these findings, she was diagnosed with grade-3 ICI-associated VKH-like posterior uveitis. The ICI therapy was discontinued according to the ASCO guidelines (20). Bilateral betamethasone eye instillation, sub-Tenon’s capsule triamcinolone acetonide injection, and oral prednisolone at a dose of 30 mg were initiated. Following the initiation of ophthalmic treatment, improvement in retinal and choroidal findings was observed (**c**). Her visual acuity at the final visit was Vd = (0.9) and Vs = (0.8). Due to progression of the primary disease, she died 10 months after initiating ICI therapy.

**Figure 2:**
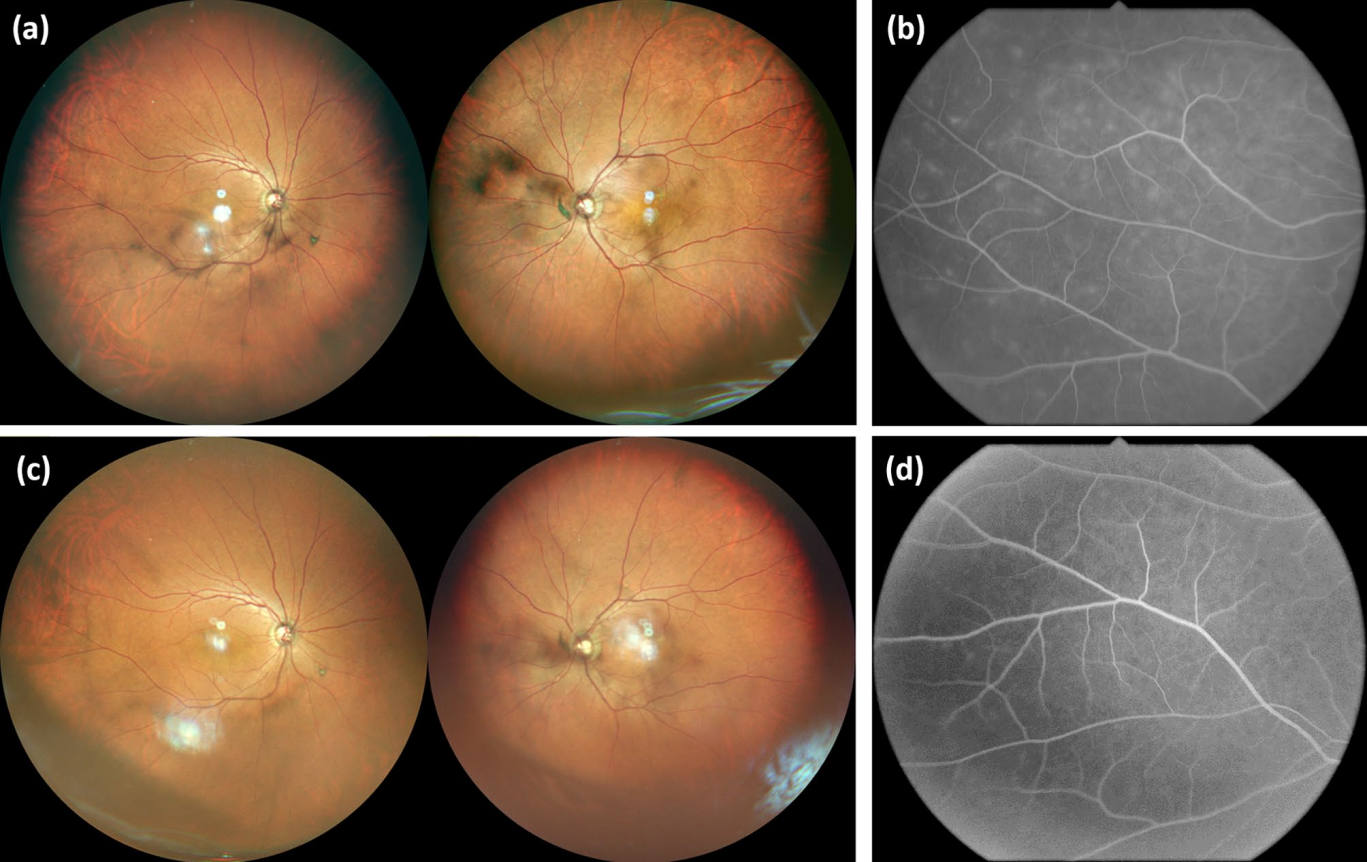
Ophthalmologic findings in a representative case of nonspecific immune checkpoint inhibitor-associated uveitis (Case 4). A 72-year-old male diagnosed with left-forearm cutaneous malignant melanoma with multiple metastases initiated treatment with nivolumab monotherapy. Although treatment with an immune checkpoint inhibitor (ICI) was effective, uveitis developed 3 months after ICI administration. His visual acuity was Vd = (1.2) and Vs = (1.2). Ophthalmologic examinations showed nonspecific inflammation of both eyes, including iridocyclitis and vitreous opacity (**a**). Fluorescein angiography detected retinal vasculitis (**b**). He was diagnosed with grade 3 ICI-associated pan uveitis. We initiated bilateral eye instillation of 0.1% betamethasone. Retinal photocoagulation was performed for a retinal tear with sub-Tenon’s capsule injection of dexamethasone. After initiating ophthalmological treatment, the anterior iridocyclitis resolved, and vitreous opacity and retinal vasculitis improved (**c**,**d**). Since his uveitis was mild and responded well to treatment, nivolumab was continued. He succumbed to progression of the primary disease 18 months after initiating the ICI therapy.

ICIU occurred bilaterally in all nine cases. Seven patients had panuveitis, and two had posterior uveitis. A total of five patients had VKH-like uveitis; four had marked thickening of the choroid and serous retinal detachment, and one had choroidal thickening and a sunsetting fundus (Fig. 1). The four cases of ICIU showed nonspecific inflammation (Fig. 2). Extraocular symptoms in VKH-like ICIU cases included hearing loss in Case 1, although it was not accompanied by tinnitus. The other four cases had no extraocular symptoms. We performed a cerebrospinal fluid examination for four VKH-like ICIU cases except Case 9 due to thrombocytopenia, and pleocytosis was not detected in any of the cases (< 5 counts/μL). IrAEs other than ICIU manifested as interstitial lung disease and thyroid dysfunction in Case 1, colitis in Case 4, and colitis and liver dysfunction in Case 7 (Table 1).

Corticosteroid eye drops were administered to treat the ICIU in all patients. One patient was cured by corticosteroid eye drops alone, while one patient had a subconjunctival injection of dexamethasone, two patients received a sub-tenon dexamethasone injection, and three patients received a sub-tenon triamcinolone acetonide injection in addition to the eye drops. Systemic treatment included 30 mg of oral prednisolone in 1 patient and 500 mg methylprednisolone pulse for 3 days in 1 patient. The local or systemic administration of corticosteroids resulted in remission or relief of uveitis in all cases. The ICI suspected to be causing the ICIU was discontinued in six cases and continued in three. ICIs were continued based on the ICIU condition and the potential benefit of the ICI in treating the primary disease. Three patients died from the primary disease during the study.

There was no observed statistical association with the disease form of ICIU and the patient age, sex, primary disease, ICI, duration of ICI administration until ICIU onset, other irAEs, clinical manifestations of uveitis, and BCVA at the first and last visits (Table 2).

**Table 2:** Comparison of clinical manifestations and HLA type according to the disease form of immune checkpoint inhibitor-associated uveitis.

	All	VKH-like	Nonspecific	*P* value
Age	65.9 ± 9.36	67.0 ± 9.32	64.5 ± 9.23	
Sex				0.21
Male	4	1	3	
Female	5	4	1	
Days to onset	74.8 ± 39.6	67.0 ± 45.5	82.5 ± 30.7	
Observation period (m)	12.7 ± 8.7	11.8 ± 10.8	13.8 ± 6.02	
ICI				1
Nivolumab	5	2	3	
Nivolumab + ipilimumab	1	1	0	
Pembrolizumab	1	1	0	
Atezolizumab	1	0	1	
Durvalumab	1	1	0	
Primary disease				1
Malignant melanoma	5	2	3	
Small-cell lung cancer	2	1	1	
Gastric cancer	1	1	0	
Gastric cancer and renal cancer	1	1	0	
Affected eye				0.44
Unilateral	1	0	1	
Bilateral	8	5	3	
Uveitis				0.44
Anterior	0	0	0	
Posterior	2	2	0	
Pan	7	3	4	
Discontinuation of ICI				1
Yes	6	3	3	
No	3	2	1	
BCVA				
Onset of ICIU	0.152 ± 0.293	0.254 ± 0.330	0.007 ± 0.136	0.19
Final visit	0.017 ± 0.132	0.043 ± 0.148	− 0.021 ± 0.094	0.48
HLA-DRB1*04:05				0.029
Positive	4	4	0	
Negative	3	0	3	

*irAE* immune-related adverse event, *ICI* immune checkpoint inhibitor, *ICIU* immune checkpoint-associated uveitis.

HLA typing was performed in seven cases for which DNA samples were obtained. Four cases with VKH-like ICIU were positive for HLA-C*01, HLA-DRB1*04:05, and HLA-DQB1*04:01 (Tables 2, 3). None of the three patients with non-VKH-like uveitis were positive for these HLA types. The frequency of HLA-C*01, HLA-DRB1 *04:05, and HLA-DQB1*04:01 was significantly higher in cases with VKH-like uveitis (*P* = 0.029). The sensitivity and specificity of HLA-C*01, HLA-DRB1 *04:05, and HLA-DQB1*04:01 were 100% and 100%, respectively. The haplotype frequency of HLA-C*01-HLA-DRB1*04:05-HLA-DQB1*04:01 in the Japanese population was 7.36%.

**Table 3:** HLA types of patients with immune checkpoint inhibitor-associated uveitis.

Case	ICIU form	HLA-A	HLA-B	HLA-C	HLA-DRB1	HLA-DQB1	HLA-DRB345
Case 1	VKH-like	A*02 A*26	B*40 B*59	C*01 C*03	DRB1*04:05 DRB1*09:01	DQB1*03:03 DQB1*04:01	DRB4*01 DRB4*01
Case 2	Nonspecific	A*26 A*26	B*40 B*40	C*03 C*07	DRB1*12:01 DRB1*14:05	DQB1*03:01 DQB1*05:03	DRB3*01:01 DRB3*02:02
Case 4	Nonspecific	A*02 A*24	B*39 B*39	C*07 C*07	DRB1*08:03 DRB1*09:01	DQB1*03:03 DQB1*06:01	DRB4*01 Blank
Case 5	VKH-like	A*02 A*26	B*39 B*46	C*01 C*07	DRB1*04:05 DRB1*09:01	DQB1*03:03 DQB1*04:01	DRB4*01 DRB4*01
Case 7	Nonspecific	A*26 A*31	B*40 B*40	C*03 C*03	DRB1*08:03 DRB1*09:01	DQB1*03:03 DQB1*06:01	DRB4*01 Blank
Case 8	VKH-like	A*24 A*24	B*52 B*59	C*01 C*12	DRB1*04:05 DRB1*04:05	DQB1*04:01 DQB1*04:01	DRB4*01 DRB4*01
Case 9	VKH-like	A*02 A*26	B*15 B*54	C*01 C*03	DRB1*04:05 DRB1*12:01	DQB1*03:01 DQB1*04:01	DRB3*01:01 DRB4*01

HLA typing was not performed for Cases 3 and 6.

*ICIU* immune checkpoint inhibitor-associated uveitis, *VKH-like* Vogt–Koyanagi–Harada disease-like uveitis.

## Discussion

The eye has an immunosuppressive mechanism called immune privilege to prevent infection and inflammation and thus maintain visual function^7^. Immune checkpoint molecules are involved in the immunosuppression process. PD-L1 and CD86, which are expressed in the retinal pigment epithelium and ocular melanocytes, bind to PD-1 and CTLA-4, respectively, to co-inhibit T-cells^1,8^. It is suggested that, in patients treated with ICIs, inhibition of the PD-L1/PD-1 and CD86/CTLA4 pathways induces ocular inflammation, causing uveitis.

The time from ICI administration to onset ICIU was 74.8 ± 39.6 days. Typical cases have been reported to develop within weeks to months, with Zhou et al. reporting a mean onset duration of 9 weeks (2 weeks–14 months)^3,9^. Anterior uveitis is more frequent than posterior uveitis and panuveitis in ICIU^4^. In this study, all patients presented with posterior inflammation and were diagnosed with posterior or panuveitis. The rate of uveitis recurrence after ICI administration in patients with a history of uveitis is reportedly as high as 51%^10^. Cases 5 and 8 who developed VKH-like ICIU had a history of VKH without recurrence until ICIU onset. VKH-like uveitis has been reported in several studies as an ocular irAE in ICI-treated patients^3,4^. A systematic review of 241 eyes in 126 patients by Dow et al., found VKH-like uveitis in 28 (35%) of 82 eyes with panuveitis^4^. In our study, 5 of 9 cases (55.6%) with ICIU had a VKH-like uveitis, with thickening of the choroid, serous retinal detachment, or sunset-like fundus and ocular manifestations of VKH. Although, extraocular symptoms, such as meningismus, tinnitus, alopecia, and poliosis, can occur in VKH-like ICIU cases as in VKH, only one case presented with sensorineural hearing loss in this study^11–13^. None of the patients showed pleocytosis on CSF examination. Although there have been few reports of CSF examination of VKH-like ICIU, pleocytosis was observed in some cases^14,15^. These findings suggest that inflammation tends to be more localized in the eye in VKH-like ICIU than in VKH.

VKH is thought to cause activation of T-cells and effector immune cells in the retinal pigment epithelial cells (RPE) and choroid via a cross-reaction between viral antigens and proteins from pigment cells^16^. Although the mechanism of VKH-like uveitis in ICI is not yet clear, it is assumed that ICIU induces a VKH-like pathogenesis due to immune abnormalities in pigment cells and RPE that express immune checkpoint molecules. VKH is more prevalent in East Asian populations than in Caucasians and is the second most common cause of uveitis in the Japanese population after sarcoidosis^17,18^. As most of the cases in the systematic review were Caucasians, the higher frequency of VKH, such as ICIU in this study of Japanese subjects may be due to differences in susceptibility by race, as well as VKH.

Treatment included local steroid therapy in all patients, oral corticosteroid therapy in one patient, and steroid pulse therapy in one patient according to the VKH treatment protocol. In cases of ophthalmic irAE, The American Society of Clinical Oncology Clinical Practice Guideline recommends the withdrawal of ICIs in Grade 2 patients with anterior uveitis and discontinuation in patients with Grade 3 or higher with posterior uveitis and severe visual loss^19,20^. In the nine patients in this study, all of whom had Grade 3 or higher ophthalmic irAE, ICIs were discontinued in six cases, and were continued in three because of the efficacy against the primary disease and the disease condition of the uveitis. In general, most ICIU patients respond well to corticosteroid therapy and have a good visual prognosis^21^. All patients in this study also had a favorable response to corticosteroid therapy and achieved relief or resolution of the inflammation without recurrence.

Regarding factors defining the clinical presentation of uveitis, no trends were found in sex, primary disease, ICI, duration of onset, or anatomic classification of uveitis. In terms of patient HLA types, 100% of patients with VKH-like uveitis who underwent HLA typing were positive for HLA-DRB1*04:05, which has also been observed in several reports of VKH-like ICIU^12,13,22–24^. DRB1*04:05 is one of the most robustly correlated risk alleles for VKH, and HLA-DR4 is positive in almost all Japanese patients with VKH^17,25^. In addition, DRB1*0405 and DRB1*0410 have been reported to be associated with prolonged VKH^26^. Since VKH-like ICIU shares the same HLA-type susceptibility as VKH, there may be a common pathogenesis with VKH. Conversely, none of the three nonspecific ICIU cases were HLA-DRB1*04:05 positive. HLA typing in non-VKH-like uveitis is rarely reported. Our study results suggest that HLA-DRB1*04:05 may be essential in defining disease form in ICIU. Further, HLA-DQB1*04:01 and HLA-C*01, which are in high LD with HLA-DRB1*04:05, were also positive in VKH-like ICIU cases and negative in non-VKH-like ICIU cases (Tables 2, 3). The HLA-C*01-HLA-DRB1*04:05-HLA-DQB1*04:01 haplotype was the second most common in the Japanese population following HLA-C*12-HLA-DRB1*15:02-HLA-DQB1*06:01 (10.0%)^6^.

The limitation of this study is that it is a single-center, retrospective study with a small number of cases. The sample size may not be sufficient to identify the factors involved in the disease form. Further studies with a larger number of cases could clarify the pathogenesis of ICIU, identify the factors involved, and establish treatment strategies. To our knowledge, most of the HLA analyses of ICIU cases to date have been case reports of VKH-like ICIU, with no reports of non-VKH-like ICIU or multiple cases. Although this study included a few cases, comprehensive HLA analysis of both VKH-like and non-VKH-like ICIUs provided new insights into the association between VKH-like ICIUs and HLA.

With continued development of ICIs in the treatment of malignancies, the incidence of ICIU uveitis will increase. This study provides evidence that the ICIU form might be influenced by the patient's HLA-DRB1 allele. In addition, the common susceptibility of HLA-DRB1*04:05 to VKH-like ICIU and VKH indicates that, in addition to similar clinical manifestations, VKH-like ICIU may have a similar pathogenesis to VKH.

## Data Availability

The data sets generated and/or analyzed in this study are available from the corresponding author upon reasonable request.
